# Higher frequency of upper gastrointestinal symptoms in healthy young Japanese females compared to males and older generations

**DOI:** 10.1007/s10388-017-0598-0

**Published:** 2017-12-19

**Authors:** Kojiro Kawachi, Yasuhisa Sakata, Megumi Hara, Eri Takeshita, Hiroharu Kawakubo, Daisuke Yamaguchi, Norihiro Okamoto, Ryo Shimoda, Ryuichi Iwakiri, Nanae Tsuruoka, Motoyasu Kusano, Kazuma Fujimoto

**Affiliations:** 1 Departments of Internal Medicine, Saga Medical School, 5-1-1 Nabeshima, Saga, 849-8501 Japan; 2 Departments of Preventive Medicine, Saga Medical School, Saga, Japan; 3 Department of Endoscopy and Endoscopic Surgery, Gunma University Hospital, Gunma, Japan

**Keywords:** Gastroesophageal reflux disease, Reflux symptoms, Acid-related dyspepsia, Frequency scale for the symptoms of GERD

## Abstract

**Background:**

The aim of this study was to evaluate the differences in upper gastrointestinal symptoms between generations and genders in relatively healthy Japanese subjects.

**Methods:**

Altogether, 4086 healthy Japanese male and female (M/F) adults (M/F: 2244/1842) were analyzed. Among them, 3505 subjects (M/F: 1922/1583) were underwent a routine medical checkup at one of five hospitals in Saga, Japan from January 2013 to December 2013. The others were 581 (M/F: 322/259) healthy young volunteers at the Saga Medical School from April 2007 to March 2013. The participants were asked to complete the frequency scale for the symptoms of gastroesophageal reflex disease (FSSG) questionnaire, undergo upper gastrointestinal endoscopy, and submit to a rapid urease test to diagnose *Helicobacter pylori* infection. Among the 4086 subjects, the 2414 who had no *H. pylori* infection and no positive endoscopic findings were enrolled in the study.

**Results:**

Subjects’ average age was 46.9 ± 12.2 years, with males’ and females’ ages being almost equivalent. The total FSSG score were high in females compared to males (*P* < 0.01) and decreased significantly with aging (*P* < 0.05). Among the generations, FSSG scores were the highest for those 20–29 years old, and they were significantly decreased with ageing in both males and females (*P* < 0.05).

**Conclusion:**

The FSSG score was significantly higher in healthy Japanese females than in males, and the scores decreased with aging.

## Introduction

Annual public health surveys conducted by the Ministry of Health, Labor, and Welfare of Japan in 2013 indicated that 27.7% of men and 31.2% of females had some type of clinical symptoms [1]. The report suggested that, among those > 20 years of age, Japanese females tended to complain more frequently than males [1]. Whereas the prevalence of abdominal symptoms in a USA population-based study showed no significant difference between genders [2] and a previous study in Japan suggested that the prevalence of non-ulcer-related dyspepsia was not different between the genders [3], most studies did not clearly demonstrate gender differences regarding upper gastrointestinal symptoms [3–5]. Our previous studies suggested that upper gastrointestinal symptoms in relatively healthy Japanese subjects were more frequent in females than in males [6, 7].

A Japanese study of the clinical signs and symptoms of gastroesophageal reflux disease (GERD) developed a frequency scale for the symptoms of GERD (FSSG). It is a questionnaire for use with Japanese subjects and covers most upper gastrointestinal symptoms [8]. The questions are related to the 12 symptoms about which the Japanese subjects complained: e.g., “heartburn” and “acidic taste” with GERD; “heavy stomach” and “feeling full quickly” in dyspeptic patients [8–11].

The present study aimed to examine; (1) whether the upper gastrointestinal symptoms evaluated by FSSG were different between relatively healthy male and female Japanese subjects, and (2) whether the tendency evaluated in these two groups was affected by aging.

## Methods

In all, 4086 male and female (M/F) healthy Japanese adults (M/F: 2244/1842) were analyzed. Among them, 3505 (M/F: 1922/1583) had undergone upper gastrointestinal endoscopy for health screening during routine medical checkups at five hospitals in Saga, Japan from January 2013 to December 2013 and were enrolled from our previous study [7]. The other 581 subjects (M/F: 322/259) were healthy volunteers (fifth-year medical students at Saga Medical School) who had undergone upper gastrointestinal endoscopy and were enrolled from a previous study conducted from April 2007 to March 2013 [6]. Those with a surgical history involving the upper gastrointestinal tract were excluded from the study. The subjects who were prescribed with the medicine which influenced the upper gastrointestinal symptoms including the gastric acid suppression medicine and/or who had the gastrointestinal disease were excluded from the analysis. We obtained informed consent from all participants. All the procedures performed in the present study were approved by the Ethical Committee of the Saga University Hospital.

All subjects (ages 24–83 years) completed an FSSG questionnaire [8] before endoscopy. *Helicobacter pylori (H. pylori)* infection was then diagnosed by the rapid urease test [12], the serum *H. pylori* immunoglobulin G antibody titer [13], and/or the urinary antibody-coated bacteria test via immunochromatography (RAPIRUN^®^; Otsuka Pharmaceutical Co., Ltd., Tokyo, Japan) [14]. The history of *H. pylori* eradication was confirmed by the patient’s medical records and history.

All subjects underwent upper gastrointestinal endoscopy, with findings of reflux esophagitis diagnosed as grade A, B, C, or D using the Los Angeles classification [15]. FSSG comprises 12 questions (7 on reflux symptoms, 5 on acid-related dyspepsia) [8]. Each symptom was assigned a score [never = 0; occasionally (30%) = 1; sometimes (50%) = 2; often (70%) = 3; always (100%) = 4]. The 12 questions were “Do you get heartburn?” “Does your stomach get bloated?” “Does your stomach ever feel heavy after meals?” “Do you sometimes subconsciously rub your chest with your hand?” “Do you ever feel sick after meals?” “Do you get heartburn after meals?” “Do you have an unusual (e.g., burning) sensation in your throat?” “Do you feel full while eating meals?” “Do some things get stuck when you swallow?” “Do you feel a bitter liquid (acid) coming up into your throat?” “Do you burp a lot?” and “Do you get heartburn if you bend over?”

**Fig. 1:**
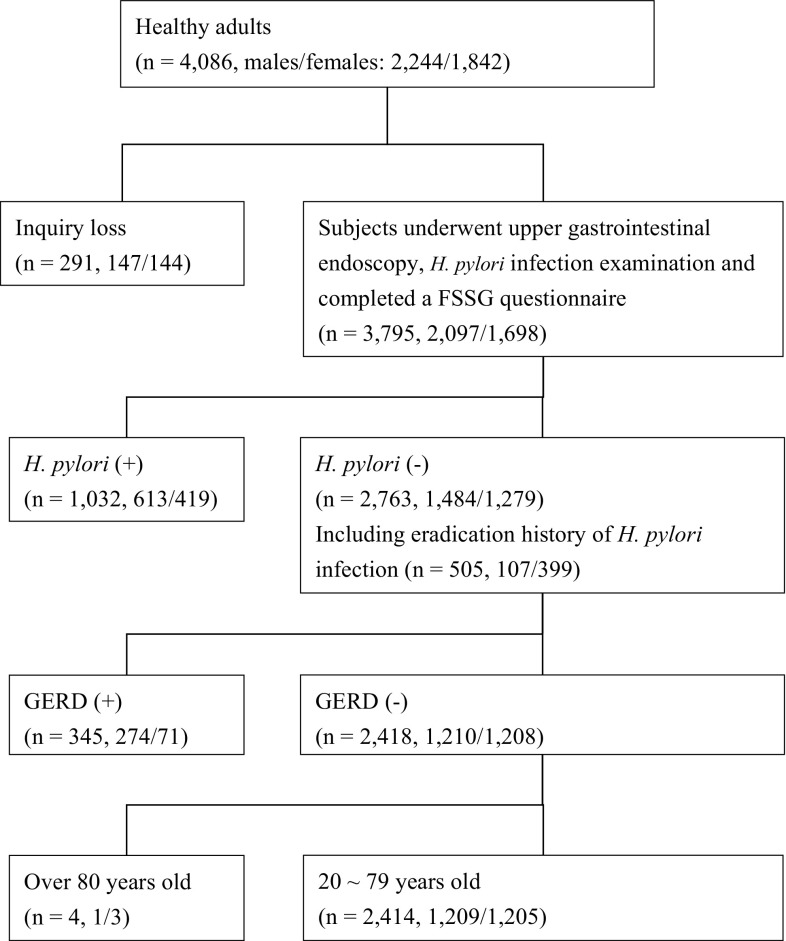
Flowchart for the selected 2414 subjects enrolled in the present study. *H. pylori Helicobacter pylori*, *Reflux* gastroesophageal reflux disease of grade A, B, C, or D

A flowchart outlining the selection process for the analyzed subjects is shown in Fig. 1. Among the 4086 healthy adults who correctly completed the questionnaires, 291 were excluded from analysis because of the inquiry loss and 1032 because of the presence of an *H. pylori* infection. Subjects who had undergone successful eradication of *H. pylori* (*n* = 505: M/F: 399/107)—504 from the medical checkup group, 1 from the medical students group—were included in the study. Among 2763 subjects, 345 with endoscopic reflux esophagitis (M/F: grade A, 236/60; grade B, 36/11; grade C, 2/0; grade D, 0/0) were excluded. Four patients > 80 years old were also excluded because of the small number of the subjects in that age group. Finally, 2414 subjects with no *H. pylori* infection and no abnormal upper gastrointestinal endoscopy findings were enrolled, as indicated in Fig. 1. The enrolled healthy subjects were then divided into six age groups (those in their 20, 30, 40, 50, 60 and 70 s).

### Statistical analysis

Descriptive statistics for continuous and categorical variables were reported as a mean with standard deviation, and as frequency and percentage, respectively. The Mann–Whitney *U* test and Kruskal–Wallis test were used to test the difference of the FSSG frequency score by gender and age groups. Pearson correlation coefficients were calculated to measure the associations for the FSSG frequency score with age group by gender. Furthermore, a multiple linear regression model was used to estimate the difference in the FSSG frequency score between age group adjusting for gender. All statistical analyses were performed with SSPS version 22 software (IBM Japan Ltd., Tokyo, Japan). *P* < 0.05 was considered to indicate a statistically significant difference.

## Results

Table 1 shows the characteristics of the healthy subjects (with no *H. pylori* infection and no endoscopic findings of reflux esophagitis). The average age was 46.9 ± 12.2 years. The ages of males and females were almost equivalent. Table 2 shows FSSG scores of the subjects in each age and gender group. Associations between age group and FSSG frequency score were significantly independent (*P* < 0.05). The total FSSG scores were significantly high in females compared to males (*P* < 0.01). The FSSG scores of those in their 20 s (20–29 years) were higher than those for any of the other generations. In addition, the FSSG score was higher in the young generations, with the score decreasing significantly along with aging. This decrease was statistically significant in the overall group of subjects (males + females) (*β*: − 1.58, *P* < 0.05), and was reflected in the significant decrease in the FSSG score with aging in females and males (*P* < 0.05). Both generation and gender were significant predictors of the FSSG score.

**Table 1:** Characteristics of subjects (*n* = 2414) without *Helicobacter pylori* infection or positive upper gastrointestinal endoscopy findings

Characteristic	Value
Age (years)	46.9 ± 12.2
Gender (males/females)	1209:1205
Body mass index (kg/m^2^)	22.6 ± 3.51

Results are given as the mean ± SD or the number

**Table 2:** FSSG scores in subjects without *Helicobacter pylori* infection or positive upper intestinal endoscopy findings, by generations (*n* = 2414)

Age (years)	Total	Males	Females
20–29	4 (2–8) (*n* = 301)	3 (1–7) (*n* = 163)	5 (2–9) (*n* = 138)
30–39	2 (0–6) (*n* = 285)	3 (1–6) (*n* = 123)	2 (0–5) (*n* = 162)
40–49	2 (0–6) (*n* = 691)	2 (0–5) (*n* = 341)	2 (0–6) (*n* = 350)
50–59	2 (0–5) (*n* = 806)	1 (0–4) (*n* = 408)	2 (0–5) (*n* = 398)
60–69	2 (0–4) (*n* = 297)	2 (0–4) (*n* = 154)	1 (0–5) (*n* = 143)
70–79	0 (0–2) (*n* = 34)	0 (0–1) (*n* = 19)	0 (0–5) (*n* = 15)
*P* for trend	< 0.05**	< 0.05*	< 0.05*

Results are given as the median (range). The Mann–Whitney *U* test and Kruskal–Wallis test were used to test the difference of the FSSG frequency score by gender and age groups

*GERD* gastroesophageal reflux disease, *FSSG* frequency scale for the symptoms of GERD

*Pearson correlation coefficients were calculated to measure the associations for the FSSG frequency score with age group by gender

**Multiple linear regression model was used to estimate the difference in the FSSG frequency score between age group adjusting for gender

Tables 3 and 4 show the influence of the age and gender on the reflux symptoms and acid-related dyspepsia scores, respectively. Associations between age group and FSSG frequency score were significantly independent (*P* < 0.05). As indicated in Table 3, the decrease in the scores with aging was statistically significant in the acid-related dyspepsia score (*β*: − 2.11, *P* < 0.05) and the reflux symptoms score (*β*:− 0.77, *P* < 0.05). The age-related decrease was significant when the analysis was performed separately by gender (i.e., in males and females separately) except the dyspepsia in males (Table 4).

**Table 3:** Reflux symptoms scores and acid-related dyspepsia scores in subjects without *Helicobacter pylori* infection or positive upper gastrointestinal endoscopy findings

Age (years)	Parameter	
	Reflux symptoms	Acid-related dyspepsia
20–29 (*n* = 301)	1 (0–3)	3 (1–5)
30–39 (*n* = 285)	1 (0–2)	2 (0–3)
40–49 (*n* = 691)	1 (0–3)	1 (0–3)
50–59 (*n* = 806)	0.5 (0–2)	1 (0–2)
60–69 (*n* = 297)	1 (0–2)	0 (0–2)
70–79 (*n* = 34)	0 (0–1)	0 (0–1)
*P* for trend*	< 0.05	< 0.05

Results are given as the median (range). The Mann–Whitney *U* test and Kruskal–Wallis test were used to test the difference of the FSSG frequency score by gender and age groups

*Multiple linear regression model was used to estimate the difference in the FSSG frequency score between age group adjusting for gender

**Table 4:** Reflux symptoms scores and acid-related dyspepsia scores in subjects without *Helicobacter pylori* infection or positive upper gastrointestinal endoscopy findings

Age (years)	Males		Females	
	Reflux	Dyspepsia	Reflux	Dyspepsia
20–29 (*n* = 301)	1 (0–3)	2 (1–4)	1 (0–4)	3.5 (1–6)
30–39 (*n* = 285)	1 (0–2)	1 (0–3)	1 (0–3)	2 (0–3)
40–49 (*n* = 691)	0 (0–3)	1 (0–3)	1 (0–3)	1 (0–3)
50–59 (*n* = 806)	1 (0–2)	0 (0–2)	0 (0–3)	1 (0–3)
60–69 (*n* = 297)	1 (0–2)	0 (0–2)	0 (0–2)	0 (0–2)
70–79 (*n* = 34)	0 (0–0)	0 (0–0)	0 (0–1)	1 (0–2)
*P* for trend*	< 0.05	0.06	< 0.05	< 0.05

Results are given as the median (range)

*Reflux* reflux symptoms score, *Dyspepsia* acid-related dyspepsia symptoms score

*Pearson correlation coefficients were calculated to measure the associations for the FSSG frequency score with age group by gender

## Discussion

Our previous studies indicated that, among relatively healthy subjects, females complained of upper gastrointestinal symptoms more commonly than males in the younger generations [6, 16] and middle-aged to older generations [7, 17]. The present study indicated that: (1) among relatively healthy subjects, FSSG-identified upper gastrointestinal symptoms were more common in females than males; (2) complaints of upper gastrointestinal symptoms diminished concomitantly with aging; and (3) age-related changes in symptoms were typical both in those with acid-related dyspepsia and reflux symptoms.

A previous study of Dutch and Japanese working populations indicated that women complained of upper gastrointestinal symptoms more often than men [18]. Other studies showed that functional dyspepsia patients in young generations of Korean and Japanese populations complained of more serious upper gastrointestinal symptoms than did aged populations [19–22]. An Internet survey in Japan of 15,000 subjects indicated that it was young females who most complained of acid-related dyspepsia [23]. The results of these reports—similar to the results of the present study—indicated that upper gastrointestinal symptoms were most significant in the young females. These results suggest that young females might overstate the upper gastrointestinal symptoms even in the clinical situation.

Several studies indicated that functional dyspepsia was more common in older and/or aged women in both Japan [24] and Western countries [25], whereas in Taiwan functional dyspepsia was more common in the younger generations [26]. The upper gastrointestinal symptoms were sometimes accompanied by other clinical symptoms, including headache [27] and gastric dysfunction [28–32] in an age-related manner. Judging from these data and the present results, complaints of the upper gastrointestinal symptoms occurred more often in young females, although they might not portend as serious a situation as when they occur in older and/or aged generations.

The present study indicated that relatively healthy young females complained of upper gastrointestinal symptoms more often than males overall and females of other generations, as shown by the FSSG questionnaire.
